# Protective Role of Donor KIR B Haplotype in Cytomegalovirus Reactivation Following T-Cell-Depleted Hematopoietic Stem Cell Transplantation

**DOI:** 10.3390/cells15151409

**Published:** 2026-08-04

**Authors:** Rehan M. Faridi, Nazanin Vaziri, Mohammad Saad Husain, Poonam Dharmani-Khan, Amit Kalra, Noureddine Berka, Jan Storek, Faisal M. Khan

**Affiliations:** 1 Cumming School of Medicine, University of Calgary, 3330 Hospital Drive NW, Calgary, AB T2N 4N1, Canada; 2 Alberta Precision Labs, Calgary, AB T2N 5G2, Canada; 3 Alberta Health Services, Calgary, AB T2N 4N1, Canada

**Keywords:** allogeneic hematopoietic cell transplantation, natural killer cells, killer Ig-like receptors, cytomegalovirus

## Abstract

**Background**: Cytomegalovirus (CMV) reactivation is a major complication after hematopoietic stem cell transplantation (HSCT). Natural killer (NK) cells help control CMV through killer-cell immunoglobulin-like receptors (KIRs) and their HLA ligands, but donor-derived CMV-specific T-cells may confound the interpretation of NK-mediated effects. **Methods**: We analyzed 276 HLA-matched (10/10) adults receiving ATG-based T-cell-depleted myeloablative HSCT with a known donor and recipient CMV serostatus. The donor and recipient KIR genotypes were scored by the Cooley B-content score (0–4; ≥2 = high). Clinically significant CMV reactivation (plasma viral load > 25,000 IU/mL, the institutional threshold for pre-emptive therapy) was analyzed with Fine–Gray competing-risks regression, stratified by the donor–recipient serostatus. **Results**: In seronegative-donor/seropositive-recipient (D−R+) pairs (n = 68), a high donor KIR B-content score was associated with a significantly lower reactivation risk (sub-hazard ratio, 0.46; 95% CI, 0.24–0.91; *p* = 0.024). No effect was seen in D+R+ pairs (n = 82; SHR, 0.65; *p* = 0.241); D+R− (n = 28) had too few events to model. A donor Tel-AA/recipient Tel-B+ mismatch was independently associated with a higher reactivation risk (adjusted HR, 2.41; 95% CI, 1.33–4.37; *p* = 0.004). The overall survival was unaffected in either stratum. **Conclusions**: A high donor KIR B-content score protects against CMV reactivation in D−R+, but not D+R+, HSCT recipients, consistent with NK dominance when CMV-specific donor T-cells are sparse. A specific donor–recipient telomeric mismatch independently modifies the risk. Donor KIR profiling warrants prospective evaluation in donor-selection algorithms.

## 1. Introduction

Cytomegalovirus (CMV) reactivation is a common and serious complication after hematopoietic stem cell transplantation (HSCT), increasing the morbidity and mortality once it progresses to disease. CMV is a double-stranded DNA herpesvirus [1] that establishes a lifelong latency in most adults [2,3]. For most individuals, CMV remains latent and asymptomatic; however, in immunocompromised or congenitally infected hosts, viral reactivation can become life-threatening [1]. Reactivation, here defined as the CMV DNAemia exceeding an institutional threshold, triggers pre-emptive antiviral therapy [4].

Despite effective monitoring, CMV remains one of the most frequent opportunistic infections after HSCT, with the mortality approaching 60% in untreated cases [5]. Affected patients also face increased risks of bacterial or fungal co-infections, graft-versus-host disease (GVHD), and non-relapse mortality [5]. Although the mortality has declined since the 1970s, the current strategies emphasize prevention rather than therapy [6]. As an early mediator of the graft-versus-leukemia effect, NK-cell reactivity is believed to influence these outcomes. NK-cells reconstitute rapidly after transplantation and exert cytotoxicity regulated by activating and inhibitory killer-cell immunoglobulin-like receptors (KIRs) [7]. We therefore explored whether immunogenetic markers, particularly donor KIR gene profiles, are associated with reduced CMV complications.

The KIR gene complex is broadly classified into A and B haplotypes. Group A haplotypes include a single activating KIR gene (KIR2DS4), whereas group B haplotypes contain up to four additional activating KIR genes (KIR2DS2, 2DS3, 3DS1, and 2DS5) [8]. The ligands for these KIRs are polymorphic HLA class-I epitopes of the classical HLA-Class I antigens, including allele groups C1/C2 on HLA-C and Bw4 on HLA-A/B. The presence or absence of these ligands, in addition to their associated KIRs, collectively determines the NK-cell reactivity.

Because donor seropositivity introduces CMV-specific memory T-cells that may confound the NK-cell effects, our analysis emphasizes seronegative donors. Previous studies have confirmed the association of various KIR genotypes with transplant-related outcomes, such as relapse [9], the incidence of a second malignancy and the incidence of acute and chronic GvHD [8]. Unlike prior studies that evaluated the KIR effects on CMV reactivation, which often included a mixed donor serostatus and lacked in vivo or ex vivo T-cell depletion, this study was designed to isolate NK-cell-mediated antiviral immunity. By focusing on in vivo T-cell-depleted HSCT recipients and specifically examining D−R+ donor–recipient pairs, we aimed to minimize the confounding from donor-derived CMV-specific memory/effector T-cells that may otherwise influence viral control. Here, we evaluate an aggregate-activating KIR model through donor-activating-KIR (B-content) scoring, allowing for an assessment of the cumulative NK-cell activation potential. A clearer understanding of these relationships might refine donor selection to improve the transplant outcomes.

## 2. Methods

### 2.1. Study Cohort

We analyzed a consecutive series of 281 HLA-matched (10/10) donor–recipient pairs who underwent ATG-based T-cell-depleted myeloablative HSCT for hematologic malignancies between Dec-2004 and Dec-2012. Four pairs were excluded for a missing donor or recipient CMV serostatus, and one pair was excluded due to follow-up < 30 days, leaving 276 pairs in the analysis cohort. Of these, 149 were matched siblings and 127 were matched unrelated donors. The recipient and donor CMV serostatus was determined by pre-transplant CMV IgG testing. The serostatus groups included D+R+ (n = 82), D+R− (n = 28), D−R+ (n = 68), and D−R− (n = 98). The D−R− pairs were included in the cohort description and in the competing-risks framework as a low-risk comparator, but were not the primary subjects of the KIR-effect analyses because CMV reactivation in this stratum is expected to arise from sources unrelated to the donor or recipient CMV history. Primary analyses of the donor KIR effects were performed within the D−R+ stratum and, as a positive control for the T-cell hypothesis, within the D+R+ stratum. This study was approved by the University of Calgary Research Ethics Board (ethics ID: REB15-1143; initial approval, 18 August 2015; most recent renewal, 18 October 2025).

### 2.2. CMV Surveillance and Disease Prevention

All CMV-seropositive recipients were managed with a pre-emptive antiviral strategy; universal antiviral prophylaxis was not used. The transplants in this cohort predated the routine adoption of letermovir prophylaxis at our centre, and all patients were therefore managed under the pre-emptive paradigm. The CMV DNAemia (plasma viral load) was determined weekly until day +100 using a polymerase chain reaction (PCR) assay (RealStar CMV PCR, Altona Diagnostics, Hamburg, Germany). Pre-emptive antiviral therapy (typically valganciclovir) was initiated when the viral load exceeded 25,000 IU/mL plasma, in accordance with the institutional protocol [10]. The primary outcome for this study was clinically significant CMV reactivation, defined as a CMV DNAemia exceeding this 25,000 IU/mL threshold.

### 2.3. KIR Genotyping

The tight cluster of the KIR genes is present on chromosome 19q13.4, which contains up to 16 KIR genes arranged in tandem and organized into A and B haplotypes [11]. The KIR genotype profiles (16 loci on chromosome 19q13.4) of the donors and recipients were obtained using a Luminex-based reverse SSO PCR assay (One Lambda, Thermo Fisher Inc. West Hills, CA, USA). The hybridized products were read on a Luminex 200 flow analyzer (Luminex Corporation. Austin, TX, USA) and interpreted with the Immuno-Polymorphism Database (IPD-KIR) reference panel [12]. HLA typing (HLA-A, -B, -C, -DRB1 and -DQB1 at a high resolution) was extracted from patient medical records and had been obtained by an equivalent SSO-based assay. Recipient HLA-C epitopes were classified into C1/C2 groups; HLA-B and -A epitopes were classified according to the Bw4/Bw6 status.

Based on the presence or absence of individual KIR genes, both the donor and recipient profiles were classified into group A and group B genotypes for the centromeric (Cen) and telomeric (Tel) clusters. Each individual received a Cooley B-content score (0–4) counting the number of B-defining regions across both parental haplotypes [13]. Donor B-content was the primary exposure of interest. Because only four donors had a B-content score ≥ 3, the scores were dichotomized as <2 vs. ≥2 for the regression analyses; a limitation of this categorization is that a dose–response relationship between the activating-KIR content and CMV reactivation could not be assessed (discussed below). Donor–recipient KIR mismatch variables were derived from the paired haplotype classifications and included the overall haplotype mismatch (donor A/A vs. recipient B/x and the reverse), the centromeric-group mismatch (donor Cen-A/A vs. recipient Cen-B+ and the reverse), the telomeric-group mismatch (donor Tel-A/A vs. recipient Tel-B+ and the reverse), and specific directional combinations (e.g., donor Tel-A/A with recipient Tel-B+). Missing-KIR-ligand analyses used the recipient HLA-C, Bw4 and A*03/A*11 status against donor KIR gene presence to identify the configurations in which the recipient lacked a cognate ligand for a donor inhibitory or activating KIR.

### 2.4. Statistical Analysis

Cumulative incidence functions for CMV reactivation above the threshold were estimated using the Aalen–Johansen non-parametric estimator, treating relapse, a second malignancy, graft failure and death without CMV reactivation as competing events. Group comparisons of the cumulative incidence functions were tested with Gray’s test. Associations between the KIR variables and the incidence of clinically significant CMV reactivation were tested with Fine–Gray sub-distribution hazard regression [14]. All applicable KIR and HLA variables were assessed in univariate analyses, together with the clinical and demographic covariates. Variables with *p* < 0.05 in univariate testing, and covariates recognized as confounders in the transplant literature [15], were entered into the multivariate model. The final Fine–Gray model included the patient age (≥45 vs. <45 years), donor type (sibling vs. unrelated), graft source (PBSC vs. BM), and significant acute graft-versus-host disease (grades II–IV) occurring before CMV reactivation. Patients whose follow-up ended before day +100 contributed person-time up to their censoring date and were treated as event-free for the interval observed; the competing-risks framework accounts for informative lost to follow-up. Cross-validation using cause-specific Cox proportional hazards models yielded concordant conclusions [16]. Two-sided *p*-values < 0.05 were considered statistically significant. All Fine–Gray analyses were performed in Stata v17 (StataCorp, College Station, TX, USA); the Aalen–Johansen cumulative-incidence estimation and cause-specific Cox models were performed in Python 3.10 using the lifelines library.

## 3. Results

### 3.1. Cohort Characteristics

The final analysis cohort comprised 276 HLA-matched (10/10) donor–recipient pairs distributed across four CMV-serostatus strata, including D+R+ (n = 82), D+R− (n = 28), D−R+ (n = 68) and D−R− (n = 98), with matched siblings (54%) and matched unrelated donors (46%) represented roughly equally (Table 1). Two features of the cohort were relevant to the analytic strategy. First, the cohort was homogeneous in terms of the graft source and conditioning: essentially, all pairs received peripheral blood stem cell grafts, and every patient received ATG-based conditioning (~69% with TBI), supporting the treatment of these variables as background rather than as major confounders. Second, the donor KIR haplotype and B-content-score distributions in the full cohort and the D−R+ subset (Supplementary Table S1) were similar to each other and to published frequencies from North American populations of predominantly European ancestry, supporting the representativeness of the cohort for immunogenetic analyses [17,18]. Significant acute GVHD occurred before CMV reactivation in 43.5% of the pairs and was adjusted for in all multivariate models.

### 3.2. Donor Activating KIRs Protect Against CMV Reactivation in D−R+ Recipients

**Figure 1:**
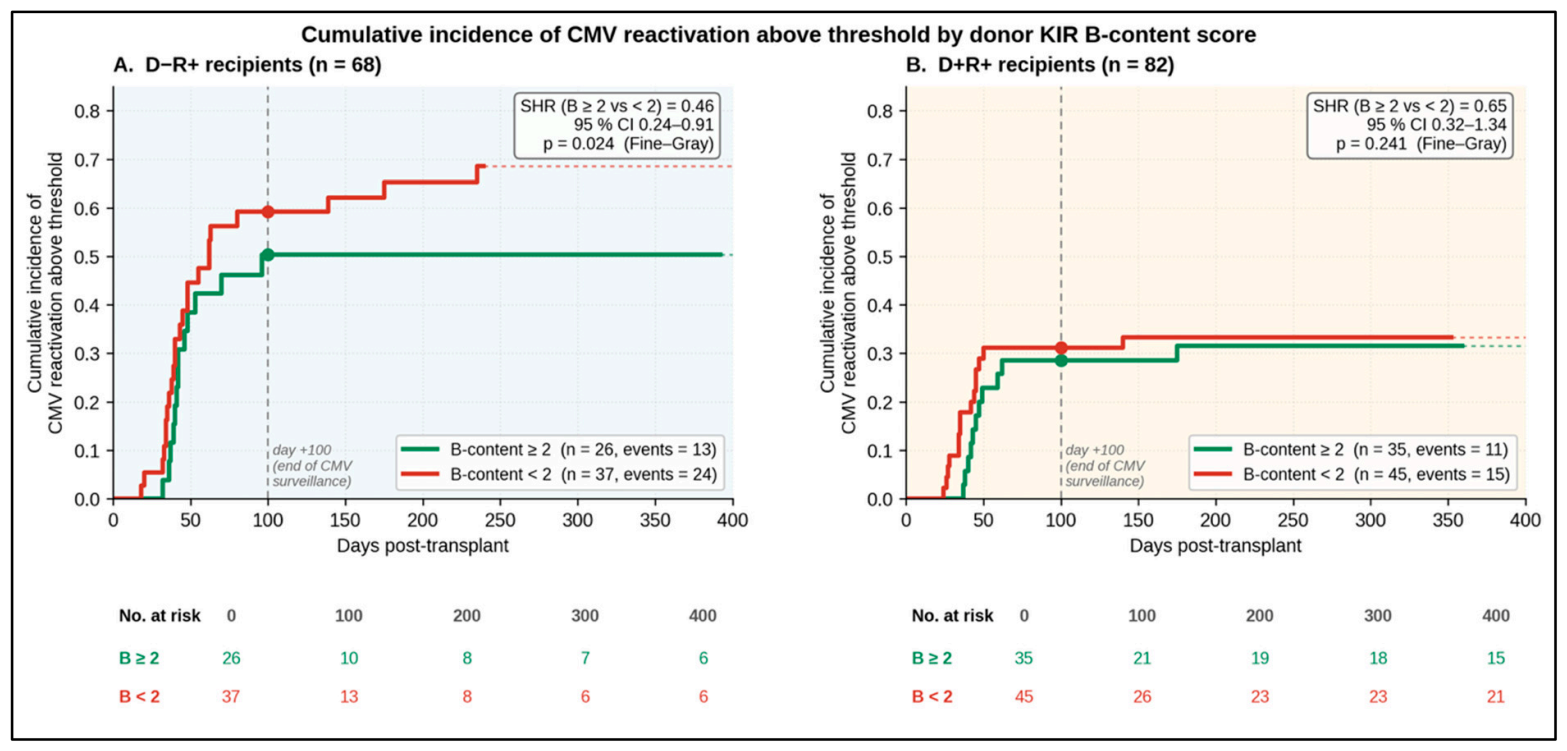
Cumulative incidence of CMV reactivation above threshold. Aalen–Johansen cumulative-incidence functions for clinically significant CMV reactivation (plasma CMV DNAemia > 25,000 IU/mL) in (**A**) D−R+ recipients (n = 68) and (**B**) D+R+ recipients (n = 82), stratified by donor KIR B-content score ≥ 2 (green) versus <2 (red). Competing events were relapse, second malignancy, graft failure and death without CMV reactivation. Vertical dashed line marks day +100 (end of CMV surveillance), and coloured dots on each curve indicate cumulative-incidence value at that timepoint. Number-at-risk tables are shown beneath each panel at days 0, 100, 200, 300 and 400. Sub-hazard ratios (SHRs), 95% confidence intervals and *p*-values are from Fine–Gray competing-risks regression adjusted for patient age (≥45 vs. <45 years), donor type (matched sibling vs. matched unrelated), graft source (PBSC vs. bone marrow) and significant acute GVHD before CMV reactivation. A donor KIR B-content score ≥ 2 was significantly protective in D−R+ recipients, but not in D+R+ recipients.

Clinically significant CMV reactivation occurred in 71 pairs overall (25.7%) and was concentrated in the D−R+ cohort, where 42 of the 68 recipients (61.8%) had reactivation above the threshold, compared with 26 of 82 (31.7%) in the D+R+, 2 of 28 (7.1%) in the D+R−, and 1 of 98 (1.0%) in the D−R− pairs. Within the D−R+ group, donors with a KIR B-content score ≥ 2 conferred a significantly lower cumulative incidence of CMV reactivation (Figure 1A). The day +100 cumulative incidence was 50% in the recipients of grafts with a B-content ≥ 2 versus 59% in the recipients with a B-content < 2. In the Fine–Gray multivariate model adjusted for patient age, donor type, graft source and significant acute GVHD before CMV reactivation, a high donor KIR B-content was associated with a sub-hazard ratio of 0.46 (95% CI, 0.24–0.91; *p* = 0.024; Table 2).

In contrast, the same predictor was not associated with CMV reactivation in D+R+ recipients (Figure 1B; SHR, 0.65; 95% CI, 0.32–1.34; *p* = 0.241; Table 2). The D+R− pairs contributed only two events in 28 patients and could not be modelled; the crude cumulative incidence at day +100 was 7.1%. In the full analytical cohort (D−R+, D+R+ and D+R− combined; n = 178), no single donor KIR grouping, including donor A/A vs. B/x, centromeric-A vs. centromeric-B, telomeric-A vs. telomeric-B, continuous B-content score, or B-content ≥ 2, reached statistical significance after adjusting for the standard covariates and donor CMV serostatus (Table 3).

### 3.3. A Donor–Recipient Telomeric KIR Mismatch Independently Increases CMV Reactivation Risk

Individual activating and inhibitory KIR genes, and their combinations with cognate HLA ligands, were tested in univariate Fine–Gray models (Supplementary Table S2). No single KIR gene or KIR–ligand pair was independently associated with CMV reactivation at *p* < 0.05; the strongest trends were for KIR2DS1 with recipient HLA-C2 (SHR, 0.49; 95% CI, 0.24–1.01; *p* = 0.053) and KIR2DS5 with recipient HLA-C1/C2 (SHR, 0.44; 95% CI, 0.19–1.02; *p* = 0.057). The recipient HLA class-I ligand groups (C1/C1, C1/C2, C2/C2; Bw4/Bw6; A*03/A*11) and counts of missing inhibitory-KIR ligands were also not associated with reactivation.

In contrast, a specific telomeric-region KIR mismatch was consistently associated with a higher risk of CMV reactivation. In the full analytical cohort, donor telomeric-group mismatch was associated with an unadjusted hazard ratio of 2.10 (95% CI, 1.29–3.43; *p* = 0.003), with the effect driven principally by the D-Tel-AA/R-Tel-B+ combination (unadjusted HR, 2.94; 95% CI, 1.69–5.09; *p* < 0.001). The D-Tel-AA/R-Tel-B+ effect remained significant after a multivariate adjustment for patient age, donor type, graft source, significant acute GVHD before CMV reactivation, and donor CMV serostatus (adjusted HR, 2.41; 95% CI, 1.33–4.37; *p* = 0.004). Within the D−R+ stratum, the effect trended in the same direction, but did not reach statistical significance (*p* = 0.057, n = 63), consistent with limited power for the interaction analysis in the smaller subgroup.

### 3.4. Donor Activating KIRs Did Not Affect Overall Survival

**Figure 2:**
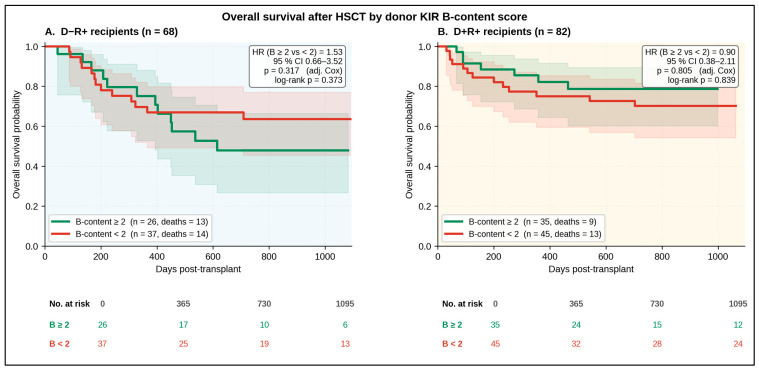
Overall survival after HSCT by donor KIR B-content score. Kaplan–Meier overall-survival curves for (**A**) D−R+ recipients (n = 68) and (**B**) D+R+ recipients (n = 82), stratified by donor KIR B-content score ≥ 2 (green) versus <2 (red). Shaded regions represent 95% pointwise confidence intervals. Number-at-risk tables are shown beneath each panel at days 0, 365, 730 and 1095. Adjusted hazard ratios (HRs), 95% confidence intervals and *p*-values are from cause-specific Cox proportional-hazard regression adjusted for patient age (≥45 vs. <45 years), donor type, graft source and significant acute GVHD before CMV reactivation; unadjusted log-rank test *p*-values are shown for reference. Donor KIR B-content score was not associated with overall survival in either the D−R+ or the D+R+ stratum.

Although high donor KIR B-content was protective against CMV reactivation in the D−R+ pairs, no association with overall survival was observed in either the D−R+ recipients (adjusted HR, 1.53; 95% CI, 0.66–3.52; *p* = 0.317; log-rank *p* = 0.373) or the D+R+ recipients (adjusted HR, 0.90; 95% CI, 0.38–2.11; *p* = 0.805; log-rank *p* = 0.839; Figure 2). The Kaplan–Meier survival curves converged early and showed wide overlapping confidence intervals in both serostatus groups, reflecting the modest number of events (13 vs. 14 deaths in D−R+; 9 vs. 13 deaths in D+R+). The dissociation between reduced early reactivation and an unchanged overall survival is discussed further below.

## 4. Discussion

### 4.1. Donor KIR B-Content Mediated CMV Regulation

In this study, a higher donor activating-KIR content, measured by a B-content score ≥ 2, was associated with a significantly reduced risk of clinically significant CMV reactivation in D−R+ HSCT recipients. This protective effect was not observed in transplants involving CMV-seropositive donors and did not translate into an improved overall survival. These findings support a model in which the cumulative activating-KIR content enhances NK-cell-mediated antiviral immunity in the early post-transplant period. By restricting the analysis to ATG-conditioned T-cell-depleted grafts and by evaluating the effect within D−R+ pairs, our study design minimized confounding from donor-derived CMV-specific memory/effector T-cells and allowed for a clearer interpretation of NK-cell-driven antiviral protection.

Prior studies have also examined the role of donor KIRs in viral reactivation, including CMV, after HSCT [19,20,21,22]. Cook et al. reported that donors with multiple activating-KIR genes had a markedly lower risk of reactivation in seropositive sibling transplants [23] and Sobecks et al. observed substantially lower reactivation with donors carrying ≥5 versus 1–4 activating-KIR genes (19% vs. 48%) [24]. Both analyses were restricted to T-cell-replete, matched-sibling settings. High donor B-content scores have also been linked to reduced relapse [25], an improved survival [26], and a lower CMV risk [26], and to reduced risks of viral reactivation in solid organ transplantation [19]. Most of these analyses emphasize individual activating-KIR genes or telomeric motifs, whereas our results support an aggregate activation model rather than the dominance of a single receptor.

### 4.2. Role of Donor Serostatus in Interpreting KIR Effects

The protective effect observed only in D−R+ transplants underscores the importance of the donor serostatus when interpreting KIR-mediated antiviral responses. Seropositive donors may transfer residual CMV-specific memory/effector T-cells, even after T-cell depletion, complicating the attribution of viral control to NK-cells alone, whereas CMV-seronegative donors provide a clearer window into NK-mediated antiviral immunity. The donor serostatus and the presence of any anti-viral T-cells must therefore be considered when interpreting immunogenetic associations with CMV reactivation after HSCT.

The D−R+ versus D+R+ contrast is itself informative. Three study design elements in our cohort pushed the T-cell influence to the background: (a) in vivo T-cell depletion with antithymocyte globulin, (b) surveillance during the first 100 days (before mature donor-derived T-cell reconstitution), and (c) the restriction of the main analysis to D−R+ pairs, which excluded the transfer of donor-derived CMV-specific memory or effector T-cells. This left D−R+ recipients largely under NK-cell-driven control, and it is precisely there that the activating-KIR effect appears. The collapse of this effect in D+R+ pairs is consistent with the dominance of donor-derived CMV-specific T-cell memory once it is available, so the D+R+ stratum acts as a positive control for the T-cell hypothesis rather than a null result. This interpretation offers a reconciliation with the findings of Cook et al., whose T-cell-replete matched-sibling cohort retained a detectable activating-KIR effect in D+R+ recipients. In T-cell-replete grafts, the donor T-cell pool is large and heterogeneous, and the CMV-specific memory fraction is relatively small, so the incremental NK contribution can remain measurable. In ATG-conditioned D+R+ transplants, memory and effector T-cell subsets appear less susceptible to depletion than naive T-cells, so the CMV-specific T-cell memory is proportionally enriched and dominates early antiviral control, masking any additional KIR contribution.

Adaptive, memory-like NK-cell responses provide a second, non-mutually exclusive explanation for the loss of the activating-KIR effect in D+R+ recipients. Cytomegalovirus is a potent driver of adaptive NK-cell expansion, generating populations expressing high levels of NKG2C together with self-KIR that display epigenetic and functional features reminiscent of memory T-cells. In seropositive donors, these adaptive NK-cells are already primed prior to donation and expand rapidly after transplantation, especially upon CMV re-exposure. Consequently, the NK-mediated antiviral response in D+R+ recipients is driven substantially by CMV-experienced adaptive NK-cells riding on the donor CMV history, rather than by the germline activating-KIR content of the graft.

### 4.3. Aggregate Activation Versus Single-Receptor Models

Unlike earlier studies implicating individual activating KIRs [19,27], we did not identify any single gene or KIR–ligand pair as being independently protective, and regional KIR genotype groupings showed only trends. These findings suggest that the overall NK-cell function may depend more on the cumulative balance between activating and inhibitory signals than on individual KIR–ligand pairs. In this context, donor B-content scoring may represent a biologically meaningful proxy for the overall activating potential of NK-cells. The contribution of individual genes is also confounded by the linkage disequilibrium between KIR loci [21,28,29], making single-gene effects difficult to isolate.

A related caveat concerns the resolution of KIR typing. Our study used sequence-specific oligonucleotide PCR at the gene-presence level, which does not resolve KIR allelic variation. This is particularly relevant for KIR2DS4, the sole activating KIR on the A haplotype, because approximately 70% of common KIR2DS4 alleles carry a 22 bp frameshift deletion (KIR2DS4*003, *004, *006, *007) [30] and do not encode a functional surface receptor. Telomeric A haplotypes are therefore heterogeneous for functional activating-KIR expression. The donor B-content score used here counts activating loci on the centromeric and telomeric B haplotypes (KIR2DS2, 2DS3, 3DS1, 2DS5) and does not incorporate KIR2DS4, so our primary analysis is robust to this issue. Nevertheless, a fuller account of the activating-KIR biology in this setting will require allele-level genotyping. Recent high-resolution KIR studies in HSCT [31,32] illustrate that allelic and haplotype-level resolution can refine associations with transplant outcomes and provide a natural direction for extending the present analyses.

### 4.4. KIR Mismatch and NK-Cell Licensing

Beyond the donor-intrinsic aggregate-activation effect, we identified a robust association between a specific telomeric KIR mismatch (donor Tel-AA with recipient Tel-B+) and higher CMV reactivation in the full cohort (adjusted HR, 2.41; 95% CI, 1.33–4.37; *p* = 0.004; Table 3, Supplementary Table S2). Because post-HSCT NK-cells are derived from donor haematopoietic stem cells and express the donor KIR repertoire, the influence of the recipient KIR status is, at first glance, counterintuitive. Two mechanisms may, however, explain this phenomenon. First, KIR and HLA loci co-evolve, so the recipient telomeric-B status is a marker of the HLA environment in which donor NK-cells are licensed after transplantation. A donor with an exclusively inhibitory telomeric profile (Tel-AA) maturing in an alternative HLA milieu shaped by the recipient telomeric-B linkage may experience disrupted licensing kinetics, yielding donor NK-cells that are hyporesponsive or functionally misdirected against CMV-infected targets.

Second, maternal microchimerism generates durable HLA and immune mosaicism in every adult recipient. Maternal-origin T, B, NK and stem cells cross the placenta during fetal life and persist in offspring for decades, at low frequencies, but in immunologically relevant tissues [33,34]. Non-inherited maternal HLA antigens (NIMAs) and persistent maternal antigen-presenting cells contribute to the “self” signal that shapes donor NK-cell education post-transplant, and residual maternal-origin NK-cells may participate in early antiviral surveillance during the window before donor NK reconstitution is complete [35]. Under this model, the recipient’s KIR haplotype does not reflect the active recipient NK function post-HSCT, but rather marks a complex, composite licensing environment comprising recipient inherited alleles, NIMAs, and persistent maternal cells that dictates donor NK-cell maturation. This telomeric mismatch effect therefore reflects a donor–recipient interaction at the level of NK-cell licensing, complementary to the donor-intrinsic B-content effect that dominates within the D−R+ analysis. Prospective studies with high-resolution KIR and HLA genotyping and NIMA-mismatch analyses will be needed to confirm this interpretation.

### 4.5. Clinical Implications and Limitations

Although a high donor B-content reduced CMV reactivation in D−R+ pairs, it did not improve the overall survival. This dissociation has two complementary explanations. First, with PCR-based surveillance and pre-emptive valganciclovir, reactivation rarely progresses to CMV disease, so the survival impact of preventing it is intrinsically limited. Second, the long-term outcomes after T-cell-depleted HSCT are shaped less by early reactivation than by the kinetics of T-cell reconstitution and by competing late events, such as relapse, GVHD, non-relapse mortality and late infections, that are largely T-cell biology and accumulate over a horizon, unaffected by the donor KIR content. The donor KIR genotype therefore acts during the window in which NK-cells dominate the antiviral defence, while survival is set by processes that unfold later through different effectors. A more speculative consideration is that CMV reactivation may itself carry a partial benefit. CMV reactivation drives the expansion of CMV-specific T-cells and adaptive NKG2C^+^ NK-cells, and some studies (though not all) have linked this expansion to reduced post-transplant relapse, an outcome described as a putative “virus-versus-leukaemia” effect [36,37]. If a high donor activating-KIR content suppresses reactivation, it may also blunt this antigenic stimulus. The non-significant trend toward better survival in B-score < 2 patients in our cohort is compatible with such an offset, although the sample size cannot resolve it.

Our study has limitations. The sample sizes within the serostatus subgroups were modest, limiting the statistical power, and the D+R− group was too small for regression modelling. Because only four donors carried a B-content score ≥ 3, the scores were dichotomized into <2 and ≥2, and a dose–response relationship between the activating-KIR content and CMV reactivation could not be assessed; larger cohorts will be needed to test whether the effect scales monotonically. The transplants in this cohort predated the routine use of letermovir prophylaxis. The demonstrated efficacy of letermovir has substantially reduced CMV reactivation in contemporary practice, and any donor-selection strategy informed by the activating-KIR content will need to be re-evaluated in cohorts receiving letermovir. Additionally, KIR genetics are inherently complex (allelic variation, copy-number differences, alternative splicing), and other NK-cell receptors, particularly NKG2C-expressing C-type lectin receptors—strongly linked to post-transplant antiviral responses—also contribute to CMV immunity [38,39,40]. Larger, integrated immunogenetic studies are needed to dissect these interactions for a more comprehensive understanding of NK-cell contributions to CMV control. The present analysis is deliberately scoped to CMV reactivation as the primary infectious endpoint. Parallel analyses of EBV reactivation and invasive fungal infections in the same cohort are the subject of companion manuscripts in preparation and will address whether the aggregate-activation and donor–recipient-mismatch effects reported here extend to non-CMV endpoints.

In conclusion, a higher donor activating KIR content, measured as a B-content score ≥ 2, was associated with a significantly reduced incidence of clinically significant CMV reactivation in D−R+ recipients of ATG-based T-cell-depleted HSCT. Consistent with the dominance of donor-derived CMV-specific memory/effector T-cells in D+R+ recipients, this effect was not observed in that stratum, nor did it translate into an improved overall survival. These findings warrant further evaluation, in larger and prospective cohorts, of whether donor activating-KIR profiling could contribute to donor-selection algorithms aimed at limiting early viral complications after HSCT.

## Figures and Tables

**Table 1:** Characteristics of study cohort.

Characteristic	Full Cohort	D+R+	D+R−	D−R+	D−R−
**N (%)**	276 (100.0)	82 (29.7)	28 (10.1)	68 (24.6)	98 (35.5)
**Donor type, n (%)**					
Matched siblings	149 (54.0)	49 (59.8)	21 (75.0)	28 (41.2)	51 (52.0)
Matched unrelated	127 (46.0)	33 (40.2)	7 (25.0)	40 (58.8)	47 (48.0)
Patient age (yr), median (range)	50 (18–66)	53 (22–66)	52 (19–66)	46 (18–66)	46 (18–66)
Patient age ≥ 45 yr, n (%)	167 (60.5)	63 (76.8)	19 (67.9)	35 (51.5)	50 (51.0)
Donor age (yr), median (range)	38 (12–68)	44 (19–67)	51 (20–64)	32 (12–66)	36 (15–68)
Donor age ≥ 45 yr, n (%)	96 (34.8)	38 (46.3)	18 (64.3)	15 (22.1)	25 (25.5)
Male donor, n (%)	179 (64.9)	50 (61.0)	13 (46.4)	47 (69.1)	69 (70.4)
**Graft source, n (%)**					
Peripheral blood stem cells	266 (96.4)	81 (98.8)	27 (96.4)	64 (94.1)	94 (95.9)
Bone marrow	10 (3.6)	1 (1.2)	1 (3.6)	4 (5.9)	4 (4.1)
**Conditioning regimen, n (%)**					
ATG-based (Flu+Bu+ATG±TBI)	276 (100.0)	82 (100.0)	28 (100.0)	68 (100.0)	98 (100.0)
Significant acute GVHD before CMV reactivation, n (%)	120 (43.5)	36 (43.9)	9 (32.1)	30 (44.1)	45 (45.9)
CMV reactivation above threshold, n (%)	71 (25.7)	26 (31.7)	2 (7.1)	42 (61.8)	1 (1.0)

Cohort: HLA-matched (10/10) donor–recipient pairs receiving ATG-based T-cell-depleted myeloablative HSCT, with ≥30 days of follow-up and known donor and recipient CMV serostatus. ATG, antithymocyte globulin; Bu, busulfan; CMV, cytomegalovirus; Flu, fludarabine; GVHD, graft-versus-host disease; PBSC, peripheral blood stem cells; TBI, total body irradiation. Threshold for CMV reactivation is CMV DNAemia > 25,000 IU/mL plasma.

**Table 2:** Donor KIR B-content score and risk of CMV reactivation.

Serostatus Stratum	n	Events	SHR (B ≥ 2 vs. <2)	95% CI	*p*
D−R+ (primary analysis)	68	41	0.46	0.24–0.91	0.024
D+R+	82	26	0.65	0.32–1.34	0.241
D+R−	28	2	—	—	—
Full analytical cohort (D−R+, D+R+, D+R−)	178	70	0.80	0.47–1.34	0.393

Fine–Gray sub-distribution hazard models adjusted for patient age (≥45 vs. <45 yr; reference < 45), donor type (matched unrelated vs. matched sibling; reference matched sibling), graft source (PBSC vs. BM; reference BM), and significant acute GVHD before CMV reactivation. Competing events: relapse, second malignancy, death without reactivation. D+R− model was not fitted because only 2 events occurred in 28 pairs (crude cumulative incidence, 7.1%). Full-cohort model additionally adjusted for donor CMV serostatus. SHR = 0.80 (95% CI, 0.47–1.34; *p* = 0.393) reflects cause-specific hazards (cross-validation).

**Table 3:** Donor KIR-genotype predictors of CMV reactivation in the full cohort.

Donor KIR Predictor	SHR (Univariate)	95% CI, *p*	HR (Multivariate Cause-Specific)	95% CI, *p*
A/A vs. B/x haplotype	0.77	0.47–1.28, 0.31	1.48	0.85–2.57, 0.16
Cen-B present vs. Cen-A/A	0.90	0.55–1.47, 0.68	0.78	0.47–1.28, 0.32
Tel-B present vs. Tel-A/A	0.87	0.53–1.42, 0.55	0.67	0.40–1.12, 0.13
Donor B-content score (continuous, 0–4)	0.93	0.72–1.19, 0.60	0.86	0.65–1.15, 0.31
Donor B-content score ≥ 2 vs. <2	0.83	0.51–1.35, 0.44	0.80	0.47–1.34, 0.39

Each row is a separate model fitted to the same analytical cohort (n = 178: D+R+ 82, D+R− 28, D−R+ 68), with 70 CMV reactivation events. Univariate: Fine–Gray sub-distribution hazards (analyst’s Stata output). Multivariate: cause-specific Cox proportional hazards adjusted for patient age (≥45), donor type, graft source, significant acute GVHD before CMV reactivation, and donor CMV serostatus (reference: D−R+).

## Data Availability

Requests for data sharing will be accommodated where possible in accordance with institutional policy and regional legislation.

## Supplementary Materials

The following supporting information can be downloaded at: https://www.mdpi.com/article/10.3390/cells15151409/s1, Table S1. Donor KIR haplotype and B-content-score distributions; Table S2. Univariate KIR–HLA, missing-ligand and mismatch predictors of CMV reactivation.
