# Supplementary material for: Protective Role of Donor KIR B Haplotype in Cytomegalovirus Reactivation Following T-Cell-Depleted Hematopoietic Stem Cell Transplantation
Source: Cells. 2026 Aug 4;15(15):1409. doi: 10.3390/cells15151409 (PMC13465379; doi:10.3390/cells15151409)
Supplement: Supplementary file 1 [file cells-15-01409-s001.zip › cells-4383521-supplementary.pdf]

Supplementary Table S1. Donor KIR haplotype and B-content-score distributions.

Genotype / category	Full cohort (n = 276)	D−R+ subset (n = 68)
**Overall donor KIR haplotype**		
A/A	65 (23.6)	14 (20.6)
B/x	195 (70.7)	49 (72.1)
**Donor centromeric group**		
Cen-A/A	108 (39.1)	27 (39.7)
Cen-A/B	126 (45.7)	26 (38.2)
Cen-B/B	26 (9.4)	10 (14.7)
**Donor telomeric group**		
Tel-A/A	147 (53.3)	34 (50.0)
Tel-A/B	99 (35.9)	24 (35.3)
Tel-B/B	14 (5.1)	5 (7.4)
**Donor KIR B-content score**		
Score 0	65 (23.6)	14 (20.6)
Score 1	100 (36.2)	23 (33.8)
Score 2	81 (29.3)	22 (32.4)
Score 3	13 (4.7)	3 (4.4)
Score 4	1 (0.4)	1 (1.5)
Score ≥ 2 (dichotomised)	95 (34.4)	26 (38.2)

Values are n (%). Denominators for percentages are the full cohort (N = 276) or the D−R+ subset (n = 68); Cen-B/B: centromeric region B-homozygous; Tel-B/B: telomeric region B-homozygous. B-content score counts the number of B-defining regions across both parental haplotypes (range 0–4). Because only 14 donors carried a B-content score ≥ 3, scores were dichotomised as < 2 vs ≥ 2 for regression analyses. Observed distributions were similar between the full cohort and the D−R+ subset and are consistent with published frequencies from North American populations of predominantly European ancestry (González-Galarza et al., 2014; Hollenbach et al., 2010).

Supplementary Table S2. Univariate KIR–HLA, missing-ligand and mismatch predictors of CMV reactivation.

Predictor	n	Events	SHR	95 % CI	p
**Donor HLA class-I ligand groups (recipient perspective)**					
HLA-C1/C1	65	25	1.00	reference	—
HLA-C1/C2	74	27	0.83	0.53–1.29	0.40
HLA-C2/C2	17	7	0.92	0.44–1.92	0.83
HLA-Bw4 present	108	44	0.94	0.60–1.48	0.79
HLA-A*03/A*11 present	70	27	0.77	0.48–1.23	0.28
**Missing inhibitory-KIR ligand (recipient)**					
Missing KIR2DL1 ligand	62	24	—	—	0.46
Missing KIR2DL2/2DL3 ligand	16	7	—	—	0.97
Missing KIR3DL1 ligand	59	20	—	—	0.61
Any missing inhibitory-KIR ligand	125	46	—	—	0.99
**Activating KIR + cognate ligand pairs**					
D_KIR2DS1 + R_HLA-C2	37	10	0.49	0.24–1.01	0.053
D_KIR2DS2 + R_HLA-C1	69	22	—	—	0.53
D_KIR2DS3 + R_HLA-C1	44	17	—	—	0.56
D_KIR2DS5 + R_HLA-C1/C2	26	5	0.44	0.19–1.02	0.057
D_KIR3DS1 + R_HLA-Bw4	35	14	—	—	0.94
**Regional KIR mismatch (donor vs recipient)**					
Overall KIR haplotype mismatch	50	18	—	—	0.64
Centromeric group mismatch	81	30	—	—	0.39
Telomeric group mismatch	67	37	2.12	1.39–3.23	< 0.001
D_Tel-AA + R_Tel-B+	24	17	3.18	1.95–5.18	< 0.001

Univariate Fine–Gray sub-distribution hazards regression, full analytical cohort (n = 178; D+R+ 82, D+R− 28, D−R+ 68). Events = CMV reactivation above the institutional threshold (25,000 IU/mL plasma; 70 events total). n is the number of pairs positive for the predictor; events is the number of CMV reactivations within that predictor group. SHR and 95 % CI are shown where the effect approaches or reaches significance; p-values only are shown where the effect is far from significance. Telomeric-group mismatch, driven principally by donor Tel-AA with recipient Tel-B-positive is reported as a secondary finding in Results §3.3 and discussed in Discussion §3b.
